# Acute hypertrophic but not maximal strength loading transiently enhances the kynurenine pathway towards kynurenic acid

**DOI:** 10.1007/s00421-020-04375-9

**Published:** 2020-04-18

**Authors:** Niklas Joisten, Moritz Schumann, Alexander Schenk, David Walzik, Nils Freitag, Andre Knoop, Mario Thevis, Wilhelm Bloch, Philipp Zimmer

**Affiliations:** 1 Department for Molecular and Cellular Sports Medicine, Institute for Cardiovascular Research and Sports Medicine, German Sport University Cologne, Cologne, Germany; 2 Center for Preventive Doping Research/Institute of Biochemistry, German Sport University Cologne, Cologne, Germany; 3 Department of “Performance and Health (Sports Medicine)”, Institute of Sport and Sport Science, Technical University Dortmund, Otto-Hahn-Str. 3, 44227 Dortmund, Germany

**Keywords:** Strenuous exercise, Acute exercise, Resistance exercise, Tryptophan metabolism, Kynurenines, Kynurenic acid

## Abstract

**Purpose:**

Due to distinct immuno- and neuro-modulatory properties, growing research interest focuses on exercise-induced alterations of the kynurenine (KYN) pathway in healthy and clinical populations. To date, knowledge about the impact of different acute strength exercise modalities on the KYN pathway is scarce. Therefore, we investigated the acute effects of hypertrophic (HYP) compared to maximal (MAX) strength loadings on the KYN pathway regulation.

**Methods:**

Blood samples of twelve healthy males (mean age and weight: 23.5 ± 3.2 years; 77.5 ± 7.5 kg) were collected before (*T*_0_), immediately after (*T*_1_), and 1 h after completion (*T*_2_) of HYP (5 sets with 10 repetitions at 80% of 1RM) and MAX (15 sets with 1RM) loadings performed in a randomized cross-over design. Serum concentrations of tryptophan (TRP), KYN, kynurenic acid (KA), and quinolinic acid (QA) were assessed using high-performance liquid chromatography.

**Results:**

The KA/KYN ratio increased from *T*_0_ to *T*_1_ (*p* = 0.01) and decreased from *T*_1_ to *T*_2_ (*p* = 0.011) in HYP, while it was maintained within MAX. Compared to MAX, serum concentrations of KA were greater in HYP at *T*_1_ (*p* = 0.014). Moreover, the QA/KA ratio was significantly lower in HYP than in MAX at *T*_1_ (*p* = 0.002).

**Conclusion:**

Acute HYP loading led to increases in the metabolic flux yielding KA, thereby possibly promoting immunosuppression and neuroprotection. Our findings emphasize the potential of acute HYP exercise as short-term modulator of KYN pathway downstream to KA in healthy males and need to be proven in other samples.

**Electronic supplementary material:**

The online version of this article (10.1007/s00421-020-04375-9) contains supplementary material, which is available to authorized users.

## Introduction

**Fig. 1:**
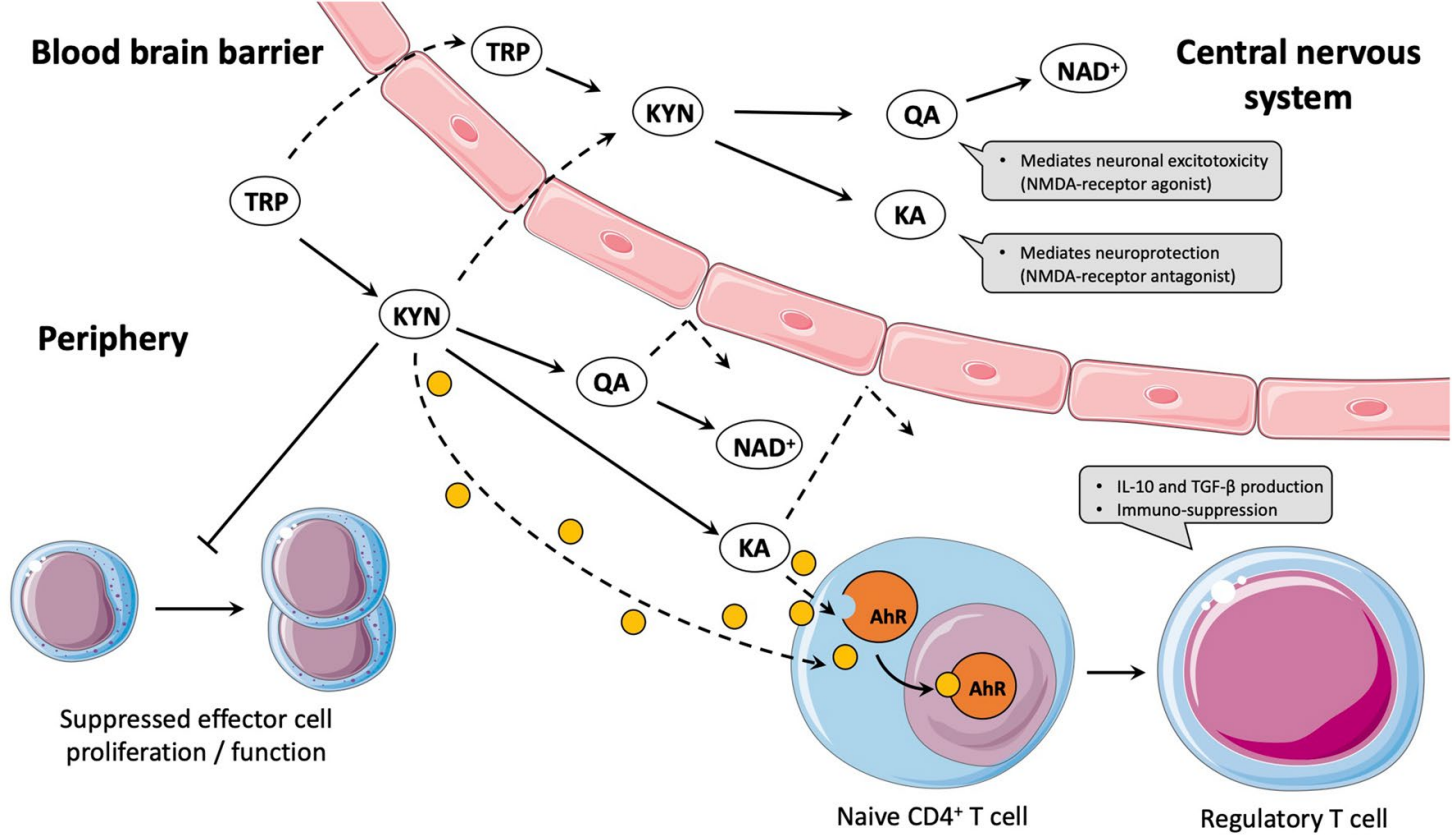
Simplified illustration of the kynurenine pathway and related immuno- and neuro-modulatory properties. While TRP and KYN can cross the blood-brain barrier and can be further degraded to neuroactive metabolites, QA and KA are not able to cross the blood-brain barrier. Concerning interactions of the KYN pathway with the immune system, KYN was described to suppress the proliferation of effector cells. Additionally, KYN and KA are ligands of the AhR, which enhances the differentiation of naive CD4^+^ T-cells to regulatory T-cells, thereby mediating immunosuppression. *TRP* tryptophan; *KYN* kynurenine, *QA* quinolinic acid; *KA* kynurenic acid; *NAD*^+^ nicotinamide adenine dinucleotide^+^; *NMDAN*-methyl-d-aspartate; *AhR* aryl hydrocarbon receptor

The essential amino acid tryptophan (TRP) plays a crucial role as precursor of different physiological processes, such as protein or serotonin synthesis as well as the kynurenine (KYN) pathway, which is closely linked to immuno- and neuro-modulatory properties. The vast majority (more than 90%) of available TRP is metabolized via the KYN pathway (Cervenka et al. 2017). While constant under basal conditions, the degradation process via the KYN pathway can dramatically increase in response to inflammatory cytokines (e.g., interferon-gamma and interleukin-6) due to the activity of the initial and rate-limiting enzyme indoleamine 2,3-dioxygenase (IDO). KYN can either be converted to kynurenic acid (KA) by the kynurenine aminotransferases (KATs) or to nicotinamide adenine dinucleotide^+^ (NAD^+^) by several intermediate steps. Detailed regulatory and functional aspects of the KYN pathway have been reviewed elsewhere (Badawy 2017). The immunomodulatory effects along the KYN pathway are primarily associated with an IDO-mediated conversion to KYN as well as with levels of KA. For example, both metabolites can promote the differentiation of T-cell subsets to anti-inflammatory regulatory T-cells (Opitz et al. 2011). Moreover, KYN has also been described to inhibit the activity of other immune cells, such as NK-cells, monocytes, and dendritic cells (Cervenka et al. 2017). Indeed, the majority of KYN is metabolized through the branch towards NAD^+^, with quinolinic acid (QA) as the most studied intermediate product (Cervenka et al. 2017). Within the central nervous system, QA strongly mediates neuronal excitotoxicity, whereas KA causes well-described neuronal protection. However, only TRP and KYN are able to cross the blood-brain barrier, while QA and KA cannot (Vecsei et al. 2013). Thus, an elevated peripheral degradation of circulating TRP and KYN levels to QA and/or KA might promote neuroprotection. Both immunosuppressive and neuroprotective properties (illustrated in Fig. 1) have led to extended research interest in the understanding and modulation of the KYN pathway in healthy and clinical populations (e.g., Parkinson’s disease, multiple sclerosis, and cancer) (Lovelace et al. 2017; Platten et al. 2019).

Recent evidence from animal and human studies suggests that both acute bouts of exercise as well as chronic training can influence the KYN pathway regulation in healthy and diseased populations (Joisten et al. 2020). The impact of skeletal muscle coactivator PGC-1α1 to regulate an exercise-induced modulation of the KYN pathway by mediating an increase in KATs was shown in a promising mice model (Agudelo et al. 2014). Indeed, Agudelo and colleagues (2014) revealed that the increase in KATs led to a peripheral KYN clearance and thereby mediated resilience to stress-induced depression. However, to the best of our knowledge, only one study in humans showed a counteracting effect of regular exercise on peripheral KYN accumulation investigating resistance exercise in breast cancer patients undergoing radiotherapy (Zimmer et al. 2019). Several other studies on regular (mainly aerobic) exercise in different populations did not observe alterations in circulating KYN pathway metabolites (Hennings et al. 2013; Kuster et al. 2017; Millischer et al. 2017). Despite exploratory studies examining different exercise modalities in multi-week interventions, a profound knowledge about the acute effects induced by single exercise bouts is warranted to allow the substantiated design of longer term interventions aiming to modulate the KYN pathway. Moreover, acute exercise-induced alterations along the KYN pathway might be involved in chronic adaptions of the immune system, e.g., via the differentiation of T-helper cell subsets towards regulatory T-cells.

Exhaustive endurance exercise has been described to induce acute alterations on KYN pathway regulation [for review, see (Metcalfe et al. 2018)], whereas the impact of acute strength exercise remains mostly unknown, especially in terms of different loading characteristics. A current study comparing acute endurance cycling and whole-body hypertrophic strength loading revealed that endurance exercise-induced larger increases in KA and QA, while only strength exercise led to a transient increase in the KYN/TRP ratio (Joisten et al. 2020). To our knowledge, no study has examined the impact of distinct strength exercise modalities on KYN pathway regulation.

Therefore, the aim of the present study was to investigate the acute response of two distinct strength loading modalities on the KYN pathway regulation. For this purpose, we replicated a previously published experimental protocol that investigated differences in immune cell and cytokine responses of a hypertrophic (HYP) and maximal strength loading (MAX) in a cross-over design (Ihalainen et al. 2014). Both types of loadings aim to improve neuromuscular characteristics through morphological (hypertrophy) or neural (maximal strength) adaptations, and represent fundamental but extreme examples of a periodized progressive strength training program, which could be beneficial for various healthy and clinical populations. Due to the greater metabolic demand and more pronounced immune responses (Ihalainen et al. 2014), we hypothesize that alterations in the KYN pathway regulation provoked by HYP will be superior to those of MAX.

## Methods

### Participants

Twelve healthy males aged between 19 and 32 years were included in this study (Table 1). Moreover, subjects were required to be experienced with regular strength training (at least one strength training session per week during the past 6 months prior to inclusion) to guarantee adequate movement control and to investigate a homogenous sample. Subjects were free of acute and chronic disease and reported not to use regular medication. The study was carried out in line with the declaration of Helsinki and was approved by the local ethics committee. Participants signed a written informed consent prior to participation.

**Table 1:** Participants’ baseline characteristics

*N* = 12	Mean ± SD
Age (years)	23.5 ± 3.2
Body height (cm)	186.8 ± 6.7
Body weight (kg)	77.5 ± 7.5
BMI (kg m^−2^)	22.3 ± 2.8
Leg press 1RM (kg)	194.6 ± 33.8
Relative leg press 1RM (kg/body weight)	2.5 ± 0.4

*SD* standard deviation, *BMI* body mass index, *1RM* one-repetition maximum

### Design and procedure

This study was a secondary analysis of a previous published study (Thamm et al. 2019), which investigated a different hypothesis using an established experimental protocol comparing acute hypertrophic and maximal strength loadings (Ihalainen et al. 2014). Accordingly, the detailed description of the pretesting and the strength loadings is reported elsewhere (Thamm et al. 2019). In brief, subjects’ anthropometric data were collected and maximal strength was assessed through a one-repetition maximum (1RM) test on a horizontal leg press device (Gym80 International GmbH, Gelsenkirchen, Germany). Subsequently, two single strength loadings (HYP and MAX, respectively) were performed in randomized order, separated by 7 days. The HYP protocol consisted of five sets with ten repetitions at 80% of 1RM with 2 min of inter-set rest. The MAX protocol consisted of 15 sets with 1 repetition at 1RM and three minutes of inter-set rest. The load was then adjusted following the initial set to achieve an actual repetition maximum. The total duration of the HYP protocol was approximately 20 min, while the MAX protocol lasted approximately 50 min. Both loadings were performed at the same time of day (07:00 am–09:00 am). Participants were asked to refrain from physical exercise 48 h prior to each testing day and were requested to avoid alcohol and caffeine intake for at least 12 h prior to each testing day. Subjects were required to maintain nutritional intake prior to each loading similar.

### Blood sampling and outcome measurements

Venous blood samples were collected before (*T*_0_) and immediately after (*T*_1_) both strength loadings as well as 1 h after completion (*T*_2_, follow-up), respectively. Blood samples were drawn from the median cubital vein by a qualified technician into serum tubes (Vacutainer, Becton Dickinson GmbH, Heidelberg, Germany). Samples were rested for 10 min and subsequently centrifugated at 3500 rpm for additional 10 min (Megafuge 3.0R., Heraeus, Germany). Thereafter, serum was aliquoted and frozen at − 80 °C until further analyses.

High-performance liquid chromatography (HPLC) and tandem mass spectrometry (MS/MS) were used to analyze serum concentrations of TRP and its metabolites KYN, QA, and KA. To indicate changes and proportions in single degradation steps along the KYN pathway, the ratios of KYN/TRP, QA/KYN, KA/KYN, and QA/KA were calculated. Sample preparation and HPLC–MS measurements were adapted from a previously published protocol (Joisten et al. 2020). Briefly, from each serum sample, two aliquots were processed in parallel. To 50 µL of serum, 5 µL of internal standard (ISTD) mixture (50 µg/mL TRP-d5, 200 ng/mL KA-d5, and 5 µg/mL QA-d3) and 25 µL of water were added. Subsequently, 70 µL of ice-cold acetonitrile were given to the sample and serum proteins were precipitated during vigorous vortexing for some seconds. To prevent analyte losses due to protein binding, samples were sonicated for 5 min before separation of the denaturated proteins by centrifugation (14,000×*g* at room temperature) as a pellet. Finally, 95 µL of the supernatant were transferred to a fresh polypropylene LC vial and fortified with 5 µL of 25% aqueous ammonia to be ready for injection to the HPLC–MS system.

The latter was composed of an ACQUITY UPLC^®^ system coupled to a Xevo^®^ TQ-XS triple quadrupole mass spectrometer, both from Waters GmbH (Eschborn, Germany). High-performance liquid chromatographic separation was achieved using a Gemini C6-Phenyl analytical column (100 mm × 2 mm ID, 3.0 µm particle size, 110 Å) from Phenomenex (Aschaffenburg, Germany) and gradient elution was carried out using 5 mM ammonium acetate (pH 9) as solvent A and acetonitrile containing 0.2% formic acid as solvent B. The overall gradient flow rate was set to 200 µL/min. Starting at 100% A, the organic phase (B) was increased in two steps to 40% and 100% within 6 and 2 min, respectively. After additional 2 min of constant 100% B, the initial starting conditions were restored and re-equilibrated for 4 min. Ionization was applied by a UniSpray™ (US) ion source operating in positive mode (US +). Individually tuned multiple reaction monitoring experiments were conducted for diagnostic ion transitions (precursor ion to product ion) resulting from collision-induced dissociations triggered by the presence of Argon: TRP *m/z* 205 → 146, KYN *m/z* 209 → 94, QA *m/z* 168 → 78 and KA *m/z* 190 → 144.

### Statistical analyses

Statistical analyses were designed to investigate outcome changes over time within (time) and between both protocols (time × group interaction). Therefore, separate univariate baseline-adjusted analysis of covariance (ANCOVA) models were conducted for each outcome. Greenhouse–Geisser correction was used in case of violations of sphericity. In case of statistical ANCOVA effects for time and/or interaction, Bonferroni corrected post hoc tests were applied. The level of significance was set at *p* ≤ 0.05. Statistical ANCOVA main effects with *p* ≤ 0.1 were accepted as a statistical trend. Cohen’s *d* effects sizes were calculated for all findings with *p* ≤ 0.1. Statistical analyses were conducted using SPSS Statistics Version 25 (IBM^®^, Armonk, NY, USA).

## Results

Participants’ anthropometric data and results from the baseline 1RM tests are presented in Table 1.

**Fig. 2:**
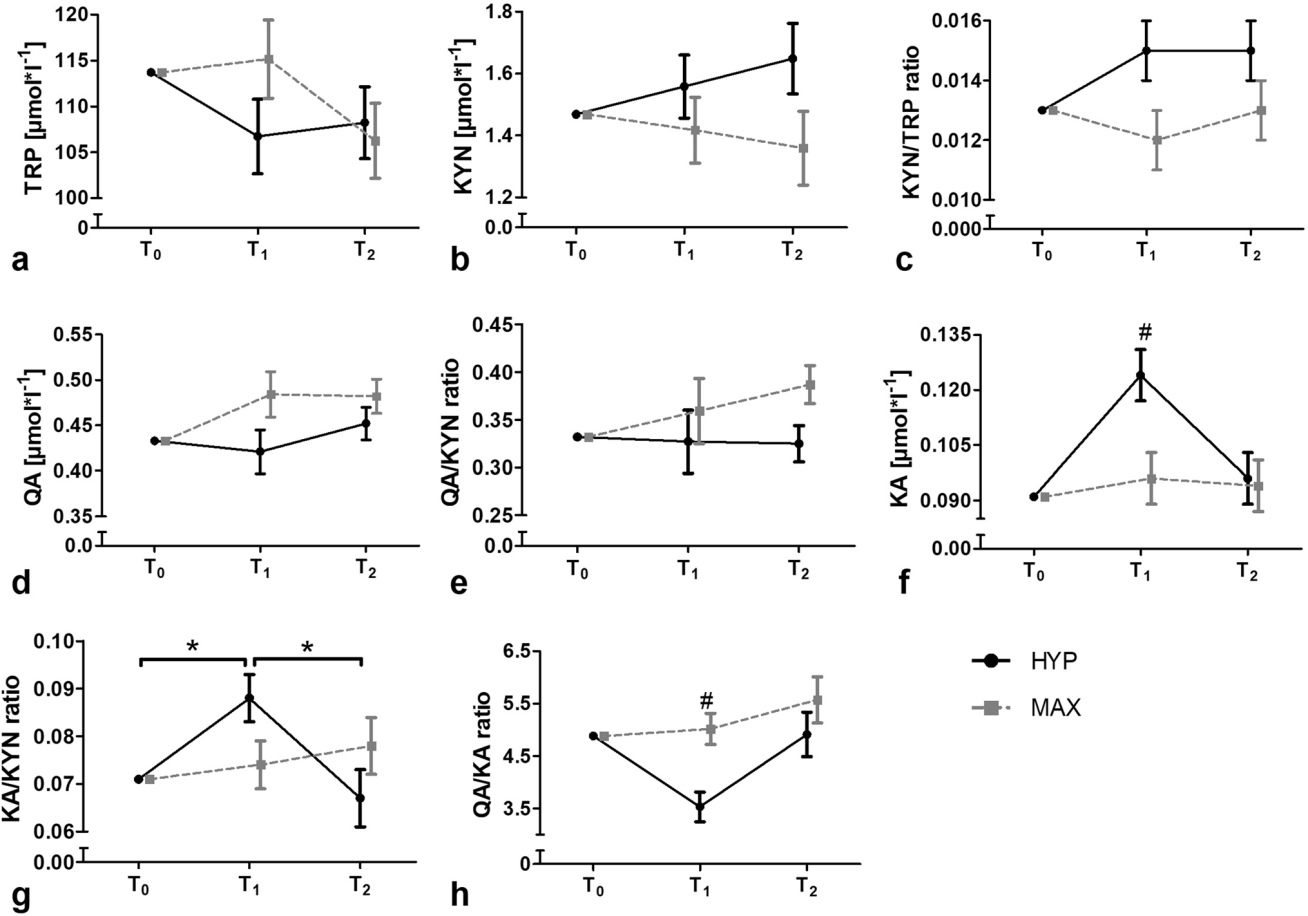
Baseline-adjusted kinetics of tryptophan and its metabolites assessed in blood serum in response to acute hypertrophic and maximal strength exercise, respectively. *Significant time effect (*p* ≤ 0.05); ^#^significant interaction effect (*p* ≤ 0.05); *HYP* hypertrophic strength loading; *MAX* maximal strength loading; *T*_0_ baseline, *T*_1_ immediately after strength loading completion; *T*_2_ 1 h after strength loading completion. **a** tryptophan concentrations; **b** kynurenine concentrations; **c** kynurenine–tryptophan ratio; **d** quinolinic acid concentrations; **e** quinolinic acid–kynurenine ratio; **f** kynurenic acid concentrations; **g** kynurenic acid–kynurenine ratio; **h** quinolinic acid–kynurenic acid ratio. Data presented as baseline-adjusted means ± standard error

Baseline-adjusted kinetics of all outcomes are shown in Fig. 2. Raw data presented as mean ± standard deviation and detailed ANCOVA results are provided in Supplement 1.

For TRP, a statistical main effect was observed for time (*p* = 0.025) but not interaction. However, the Bonferroni post hoc test did not reveal any statistical changes.

No statistically significant main effect for time and interaction was observed in KYN, the KYN/TRP ratio, QA, and the QA/KYN ratio. However, a tendency for a statistical main effect for time was observed for KA (*p* = 0.056). In HYP, the post hoc test showed a statistical increase from baseline to post-loading (*p* = 0.001) and a statistical decrease from post-loading to follow–up (*p* < 0.001). In MAX, post hoc test did not reveal any statistical changes. Moreover, in KA, a statistical effect was found for interaction (*p* = 0.009). Bonferroni post hoc test revealed a significant difference between the groups at post-loading (*p* = 0.014, *d* = − 0.555) with concentrations of KA being larger in HYP than in MAX.

For the KA/KYN ratio, statistical main effects were observed for time (*p* = 0.021) and interaction (*p* = 0.019). KA/KYN statistically increased from baseline to post-loading (*p* = 0.01) and statistically decreased from post-loading to follow-up (*p* = 0.011) in HYP, while it remained statistically unaltered in MAX. No significant differences between the groups were revealed by Bonferroni’s post hoc test. The effect sizes between the groups were *d* = − 0.382 at post-loading and *d* = 0.448 at follow-up.

For QA/KA ratio, a statistical ANCOVA effect was observed for interaction (*p* = 0.026) but not time. The loading-induced effect between HYP and MAX was observed at post-loading, with values being statistically smaller in HYP compared to MAX (*p* = 0.002, *d* = 0.728).

Calculated effect sizes for ANCOVA main effects for time with *p* ≤ 0.1 are presented in Table 2. Participants’ individual loading responses for KA, KA/KYN ratio, and QA/KA ratio are provided in Supplement 2.

**Table 2:** Cohen’s *d* effect sizes calculated for all ANCOVA main effects for time with *p* ≤ 0.1

	HYP			MAX		
	*T*_0_ → *T*_1_	*T*_1_ → *T*_2_	*T*_0_ → *T*_2_	*T*_0_ → *T*_1_	*T*_1_ → *T*_2_	*T*_0_ → *T*_2_
TRP	− 0.454	0.069	− 0.418	0.411	− 0.65	− 0.306
KA	0.915	− 0.713	0.241	0.183	− 0.015	0.16
KA/KYN	0.491	− 0.622	− 0.096	0.122	0.185	0.274

*HYP* hypertrophic strength loading, *MAX* maximal strength loading, *T*_*0*_ baseline, *T*_*1*_ post-loading, *T*_*2*_ follow-up, *TRP* tryptophan, *KA* kynurenic acid, *KA/KYN* kynurenic acid/kynurenine ratio

## Discussion

This study aimed to investigate the acute effects of two distinct strength loadings on the KYN pathway. Although we did not find a statistical alteration in the initial KYN pathway activation following either of the loadings, the downstream kinetics differed between HYP and MAX. This was especially shown by a statistical increase in KA as well as by the decrease in QA/KA ratio in HYP compared to MAX. However, these initial increases were already diminished after 1 h of recovery.

Interestingly, neither of the loadings statistically induced an initial activation of the KYN pathway, as typically indicated by decreases in TRP accompanied by increases in KYN and consequently an increase in the KYN/TRP ratio. Although a statistical main effect for time was observed for TRP, Bonferroni post hoc analysis did not indicate statistical changes. The calculated effect sizes, however, suggest a reduction in TRP in response to both loadings, albeit occurring at different time points. While the decrease in TRP concentration in HYP is emphasized from baseline to post-loading (*d* = − 0.454), the decrease in MAX was most apparent during recovery, i.e., from post-loading to follow-up (*d* = − 0.65), and was more pronounced than in HYP. Considering that no statistical increases in KYN were found in neither of the loadings, the decreases in TRP might be due to an elevated demand in amino acids for protein biosynthesis following acute strength exercise. A potential explanation for the time discrepancy of TRP reduction may represent the different duration of both exercise protocols. Moreover, differences in the time under tension and metabolic demand between both loadings as well as divergent inter-set resting periods might lead to varying recovery courses. However, the assumptions on TRP decreases are only based on effect sizes and need to be investigated in future studies with larger sample sizes.

Similar to TRP, we neither found statistical alterations in KYN nor the KYN/TRP ratio following either of the loadings. These findings are, at least partly, in contrast to results of a previous study comparing the acute effects of endurance and strength exercise on KYN pathway metabolites (Joisten et al. 2020). Interestingly, in this previous study, a statistical increase in the KYN/TRP ratio was found immediately after the strength exercise. However, the strength loading characteristics differed substantially between this previous and our current study. Joisten et al. examined the impact of a machine-based whole-body hypertrophic exercise protocol with a total duration of 50 min (Joisten et al. 2020), while in the present study, the strength loading consisted of leg press exercise only, consequently resulting in a much lower overall training volume. From a descriptive point of view, the KYN/TRP ratio in the present study also increased immediately after HYP. Compared to Joisten et al. (2020) absent statistical effects within HYP could be due to the shorter exercise duration, the lower amount of addressed skeletal muscle mass and/or the smaller sample size. Additionally, differences between loading characteristics (e.g., inter-set rest, time under tension, metabolic strain, and workload) represent potential explanations for absent statistical effects. Future studies examining acute effects of HYP on the initial KYN pathway activation should extend the exercise duration and include more measurement time points even during the exercise sessions.

Although no statistical changes in the KYN pathway branch towards QA were observed, HYP led to a transient statistical increase of the metabolic flux towards KA. This short-term modulation was primarily indicated by the statistical increase in the KA/KYN ratio from baseline to post-loading, which was subsequently followed by a statistical decrease to follow-up in HYP. Although not statistically significant in post hoc comparison, concentrations of KA in HYP also increased notably from baseline to post-loading and subsequently decreased to follow-up according to the effect sizes [*d* = 0.915 (*T*_0_ − *T*_1_), *d* = − 0.713 (*T*_1_ − *T*_2_)]. Similar results were reported in studies investigating the acute effects of endurance exercise (Lewis et al. 2010; Mudry et al. 2016). However, these findings are contrary to the results from Joisten et al. (2020), which might again be explained by different loading characteristics. It is, however, worth mentioning that both loadings assessed in the present study induce different physiological stimuli leading to distinct responses and adaptions. Whereas hypertrophic strength exercise is generally known to induce rather metabolic alterations, maximal strength exercise is characterized by the greater mechanical stimuli on skeletal muscle, albeit over a short duration. In line with the previous investigations on acute endurance exercise, metabolic stimuli seem to be more likely to impact the KYN pathway than mechanical strain. However, the present findings suggest that acute HYP transiently stimulates the KYN pathway metabolism leading to KA, thereby emphasizing the hypothesis of a peripheral KYN clearance induced by exercise. Furthermore, KA has been described as a potent endogenous ligand of the aryl hydrocarbon receptor (AhR), a well-known transcription factor with suppressive properties concerning adaptive immune responses (Murray et al. 2014; Platten et al. 2019). Thus, the transiently increased KA availability could improve AhR-mediated immunomodulation, which might be linked to the anti-inflammatory effects induced by single-bouts of exercise. This mechanism could be of major clinical relevance for persons with inflammation-driven diseases (e.g., diabetes and multiple sclerosis), especially in view of the prescription of regular hypertrophic exercise training and should, therefore, be addressed by future investigations.

Importantly, the determination of KYN metabolites in blood serum does not necessarily provide knowledge about the underlying mechanisms and the enhanced conversion of KYN to KA could be mediated by different cell types. While studies investigating the chronic effects of endurance exercise implicate that skeletal muscle may enhance KA production by PGC-1α-mediated KATs expression, this mechanistic link remains to be shown for acute exercise. Although a large body of evidence suggests PGC-1α upregulations in response to different acute exercise modalities in skeletal muscle (Baar et al. 2002; Hood 2009; Silvennoinen et al. 2015), to the best of our knowledge, there is currently only one study which assessed KAT expression after acute endurance exercise, but did not observe statistical changes (Mudry et al. 2016). In general, different PGC-1α isoforms need be considered when comparing different exercise modalities. While acute endurance exercise is known to impact the PGC-1α1 isoform in skeletal muscle, hypertrophic strength exercise is closely linked to PGC-1α4 (Ruas et al. 2012). In line with this, our results show a transiently enhanced KYN pathway branch to KA only in response to HYP. However, the mechanistic link between PGC-1α-mediated KAT expressions in skeletal muscle was exclusively shown for PGC-1α1 isoform and in response to chronic exercise (Agudelo et al. 2014). Considering that effects of both PGC-1α isoforms strongly differ (Ruas et al. 2012), it can be questioned whether observed acute increases in the conversion of KYN to KA are mediated by PGC-1α4 in skeletal muscle. Interestingly, we recently showed that KAT isoform 4 was upregulated in peripheral blood mononuclear cells immediately after acute endurance exercise (Joisten et al. 2020). However, the mechanisms of acute exercise-induced alterations in the KYN pathway regulation remain to be investigated and current evidence suggests that different cell types may be involved.

Finally, also the statistical interaction for the QA/KA ratio observed immediately post-loading emphasizes different kinetics between HYP and MAX. While the QA/KA ratio decreased at post-loading in HYP, it remained constant in MAX. Since no changes in QA and in the QA/KYN ratio were found in the present study, the shift of the QA/KA ratio towards KA in HYP is presumably driven by the increased metabolic flux of the KYN pathway yielding KA in HYP. Despite the possible neuroprotective effects induced by enhanced metabolic flux towards KA in the peripheral bloodstream, profound insights in the effects of exercise on the KYN pathway regulation within the CNS would also allow to draw conclusions on the clinical relevance. Especially for neurological diseases that are accompanied by KYN pathway disturbances (multiple sclerosis, Alzheimer’s disease), exercise-induced neuroprotective properties mediated by KYN pathway modulations would be of major importance and may contribute to improvements of various symptoms, such as cognitive impairment and/or fatigue.

### Strength and limitations

To our knowledge, this is the first study investigating the acute impact of two different strength loadings on the KYN pathway regulation. In contrast to most existing studies on acute effects of exercise that solely examined TRP and KYN as outcome measures, we also provide information on the downstream pathway kinetics. We decided to use a previously published protocol (Ihalainen et al. 2014) to build upon the reported stronger immunological changes and higher metabolic demand in HYP. From an exercise scientific point of view, targeted exercise modalities were designed to reflect two distinct stimuli, i.e., a more metabolic (HYP) as well as a more neural (MAX) stimulus. However, we acknowledge that defining these stimuli comes along with compromises in total work performed in the two loadings. Thus, a comparison of distinct loadings necessarily leads to differences in applied modalities between conditions (i.e., time under tension, inter-set rest, and total duration of training sessions) and exercise-induced strains, although representing realistic training scenarios that can be applied in various populations. This also includes different total durations of the two loadings and associated time points for the post measurements, possibly limiting the interpretation of our findings. Finally, we investigated a homogenous sample that only included young healthy males to avoid variance due to age and sex. Our findings need to be proven for female participants as well as participants of different age groups and in larger sample sizes.

## Conclusion

This study shows that acute HYP but not MAX transiently enhances the metabolic flux of the KYN pathway towards KA, thereby potentially inducing peripheral KYN clearance and immunomodulatory effects. In line with our findings, future studies investigating the effects of strength exercise on KYN pathway alterations should focus on hypertrophic strength exercise modalities. However, these findings need to be proven in other populations and in larger sample sizes. Subsequently, longer term randomized-controlled interventional studies are warranted to investigate potential chronic modulations of the KYN pathway induced by strength exercise. In perspective, strength exercise-induced modulations of the KYN pathway could be of major relevance for various clinical populations with inflammation-driven diseases, since anti-inflammatory and neuroprotective mechanisms might be initiated. Additionally, regular strength exercise stimuli that reroute the KYN pathway metabolism towards KA might also be relevant for the prevention of chronic diseases due to greater AhR ligand availability, ultimately resulting in increased anti-inflammatory properties. This hypothesis also needs to be addressed in future studies.

## Electronic supplementary material

Below is the link to the electronic supplementary material.

Supplementary file1 (DOCX 265 kb)

## Abbreviations

1RM: One-repetition maximum
AhR: Aryl hydrocarbon receptor
ANCOVA: Analysis of covariance
HPLC: High-performance liquid chromatography
HYP: Hypertrophic
IDO: Indoleamine 2,3-dioxygenase
KA: Kynurenic acid
KATs: Kynurenine aminotransferases
KYN: Kynurenine
MAX: Maximal
MS/MS: Tandem mass spectrometry
NAD^+^: Nicotinamide adenine dinucleotide^+^
NK-cell: Natural killer cell
QA: Quinolinic acid
TRP: Tryptophan

## References

[CR1] Agudelo LZ (2014). Skeletal muscle PGC-1alpha1 modulates kynurenine metabolism and mediates resilience to stress-induced depression. Cell.

[CR2] Baar K (2002). Adaptations of skeletal muscle to exercise: rapid increase in the transcriptional coactivator PGC-1. FASEB J.

[CR3] Badawy AA (2017). Kynurenine pathway of tryptophan metabolism: regulatory and functional aspects. Int J Tryptophan Res.

[CR4] Cervenka I, Agudelo LZ, Ruas JL (2017). Kynurenines: Tryptophan's metabolites in exercise, inflammation, and mental health. Science.

[CR5] Hennings A, Schwarz MJ, Riemer S, Stapf TM, Selberdinger VB, Rief W (2013). Exercise affects symptom severity but not biological measures in depression and somatization—results on IL-6, neopterin, tryptophan, kynurenine and 5-HIAA. Psychiatry Res.

[CR6] Hood DA (2009). Mechanisms of exercise-induced mitochondrial biogenesis in skeletal muscle. Appl Physiol Nutr Metab.

[CR7] Ihalainen J (2014). Acute leukocyte, cytokine and adipocytokine responses to maximal and hypertrophic resistance exercise bouts. Eur J Appl Physiol.

[CR8] Joisten N (2020). Exercise and the Kynurenine pathway: Current state of knowledge and results from a randomized cross-over study comparing acute effects of endurance and resistance training. Exerc Immunol Rev.

[CR9] Kuster OC (2017). Novel blood-based biomarkers of cognition, stress, and physical or cognitive training in older adults at risk of dementia: preliminary evidence for a role of BDNF, irisin, and the kynurenine pathway. J Alzheimers Dis.

[CR10] Lewis GD (2010). Metabolic signatures of exercise in human plasma. Sci Transl Med.

[CR11] Lovelace MD (2017). Recent evidence for an expanded role of the kynurenine pathway of tryptophan metabolism in neurological diseases. Neuropharmacology.

[CR12] Metcalfe AJ, Koliamitra C, Javelle F, Bloch W, Zimmer P (2018). Acute and chronic effects of exercise on the kynurenine pathway in humans—a brief review and future perspectives. Physiol Behav.

[CR13] Millischer V, Erhardt S, Ekblom O, Forsell Y, Lavebratt C (2017). Twelve-week physical exercise does not have a long-lasting effect on kynurenines in plasma of depressed patients. Neuropsychiatr Dis Treat.

[CR14] Mudry JM (2016). Direct effects of exercise on kynurenine metabolism in people with normal glucose tolerance or type 2 diabetes. Diabetes Metab Res Rev.

[CR15] Murray IA, Patterson AD, Perdew GH (2014). Aryl hydrocarbon receptor ligands in cancer: friend and foe. Nat Rev Cancer.

[CR16] Opitz CA (2011). An endogenous tumour-promoting ligand of the human aryl hydrocarbon receptor. Nature.

[CR17] Platten M, Nollen EAA, Rohrig UF, Fallarino F, Opitz CA (2019). Tryptophan metabolism as a common therapeutic target in cancer, neurodegeneration and beyond. Nat Rev Drug Discov.

[CR18] Ruas JL (2012). A PGC-1alpha isoform induced by resistance training regulates skeletal muscle hypertrophy. Cell.

[CR19] Silvennoinen M (2015). PGC-1 isoforms and their target genes are expressed differently in human skeletal muscle following resistance and endurance exercise. Physiol Rep.

[CR20] Thamm A, Freitag N, Figueiredo P, Doma K, Rottensteiner C, Bloch W, Schumann M (2019). Can heart rate variability determine recovery following distinct strength loadings? A randomized cross-over trial. Int J Environ Res Public Health.

[CR21] Vecsei L, Szalardy L, Fulop F, Toldi J (2013). Kynurenines in the CNS: recent advances and new questions. Nat Rev Drug Discov.

[CR22] Zimmer P (2019). Resistance exercise reduces kynurenine pathway metabolites in breast cancer patients undergoing radiotherapy. Front Oncol.

