# Supplementary material for: Acute hypertrophic but not maximal strength loading transiently enhances the kynurenine pathway towards kynurenic acid
Source: Eur J Appl Physiol. 2020 Apr 18;120(6):1429–36. doi: 10.1007/s00421-020-04375-9 (PMC7237519; doi:10.1007/s00421-020-04375-9)
Supplement: Supplementary file 1 — Supplementary file1 (DOCX 265 kb) [file 421_2020_4375_MOESM1_ESM.docx]

Supplementary Material

Acute hypertrophic but not maximal strength loading transiently enhances the kynurenine pathway towards kynurenic acid

European Journal of Applied Physiology

Niklas Joisten^1, #^, Moritz Schumann^1, #^, Alexander Schenk^1^, David Walzik^1^, Nils Freitag^1^, Andre Knoop^2^, Mario Thevis^2^, Wilhelm Bloch^1^, Philipp Zimmer^1, 3 *^

^1^ Department for Molecular and Cellular Sports Medicine, Institute for Cardiovascular Research and Sports Medicine, German Sport University Cologne, Cologne, Germany

^2^ Center for Preventive Doping Research/Institute of Biochemistry, German Sport University Cologne, Cologne, Germany

^3^ Department Exercise and Health, Institute of Sports Science, Leibniz University Hannover, Hanover, Germany

^*^Corresponding author: Dr. Dr. Philipp Zimmer, Department for Molecular and Cellular Sports Medicine, Institute for Cardiovascular Research and Sports Medicine, German Sport University Cologne, Am Sportpark Müngersdorf 6, 50933 Cologne, Germany, [p.zimmer@dshs-koeln.de](mailto:p.zimmer@dshs-koeln.de), +49 (0) 221 4982 5440

Supplement 1. Raw data presented as mean ± standard deviation and detailed ANCOVA results for all outcome measures.

|  | | Mean ± SD | | ANCOVA  time | ANCOVA  interaction |
| --- | --- | --- | --- | --- | --- |
|  |  | HYP | MAX |  |  |
| TRP  [µmol/L] | T_0_  T_1_  T_2_ | 116.99 ± 18.50  108.77 ± 17.69  109.91 ± 15.24 | 109.54 ± 16.68  118.28 ± 25.05  104.4 ± 16.89 | p=0.025  F=4.034  df=2 | p=0.154  F=1.962  df=2 |
| KYN  [µmol/L] | T_0_  T_1_  T_2_ | 1.42 ± 0.52  1.52 ± 0.64  1.6 ± 0.73 | 1.47 ± 0.07  1.44 ± 0.49  1.41 ± 0.66 | p=0.208  F=1.635  df=2 | p=0.153  F=1.965  df=2 |
| KYN/TRP | T_0_  T_1_  T_2_ | 0.012 ± 0.005  0.014 ± 0.006  0.015 ± 0.006 | 0.014 ± 0.006  0.013 ± 0.005  0.014 ± 0.006 | p=0.213  F=1.606  df=2 | p=0.109  F=2.344  df=2 |
| QA  [µmol/L] | T_0_  T_1_  T_2_ | 0.43 ± 0.17  0.42 ± 0.14  0.45 ± 0.15 | 0.42 ± 0.17  0.47 ± 0.20  0.49 ± 0.18 | p=0.228  F=1.546  df=2 | p=0.125  F=2.191  df=2 |
| QA/KYN | T_0_  T_1_  T_2_ | 0.33 ± 0.14  0.33 ± 0.2  0.32 ± 0.16 | 0.33 ± 0.2  0.35 ± 0.16  0.39 ± 0.19 | p=0.264  F=1.369  df=1.461 | p=0.27  F=1.343  df=1.461 |
| KA  [µmol/L] | T_0_  T_1_  T_2_ | 0.09 ± 0.03  0.12 ± 0.04  0.09 ± 0.03 | 0.09 ± 0.03  0.1 ± 0.03  0.1 ± 0.03 | p=0.056  F=3.094  df=2 | p=0.009  F=5.271  df=2 |
| KA/KYN | T_0_  T_1_  T_2_ | 0.069 ± 0.036  0.087 ± 0.037  0.066 ± 0.031 | 0.071 ± 0.034  0.075 ± 0.026  0.080 ± 0.034 | p=0.021  F=4.244  df=2 | p=0.019  F=4.402  df=2 |
| QA/KA | T_0_  T_1_  T_2_ | 5.178 ± 1.831  3.717 ± 1.236  5.209 ± 2.392 | 4.619 ± 1.936  4.806 ± 1.716  5.247 ± 2.399 | p=0.176  F=1.922  df=1.297 | p=0.026  F=5  df=1.297 |

HYP hypertrophic strength exercise protocol; MAX maximal strength exercise protocol; SD standard deviation; TRP tryptophan; KYN kynurenine; KYN/TRP kynurenine/tryptophan ratio; QA quinolinic acid; QA/KYN quinolinic acid/kynurenine ratio; KA kynurenic acid; KA/KYN kynurenic acid/kynurenine ratio; QA/KA quinolinic acid/kynurenic acid ratio; p p-value; F F-value; df degrees of freedom.

Supplement 2. Participants’ individual kinetics for kynurenic acid, kynurenic acid-kynurenine ratio and quinolinic acid-kynurenic acid ratio.


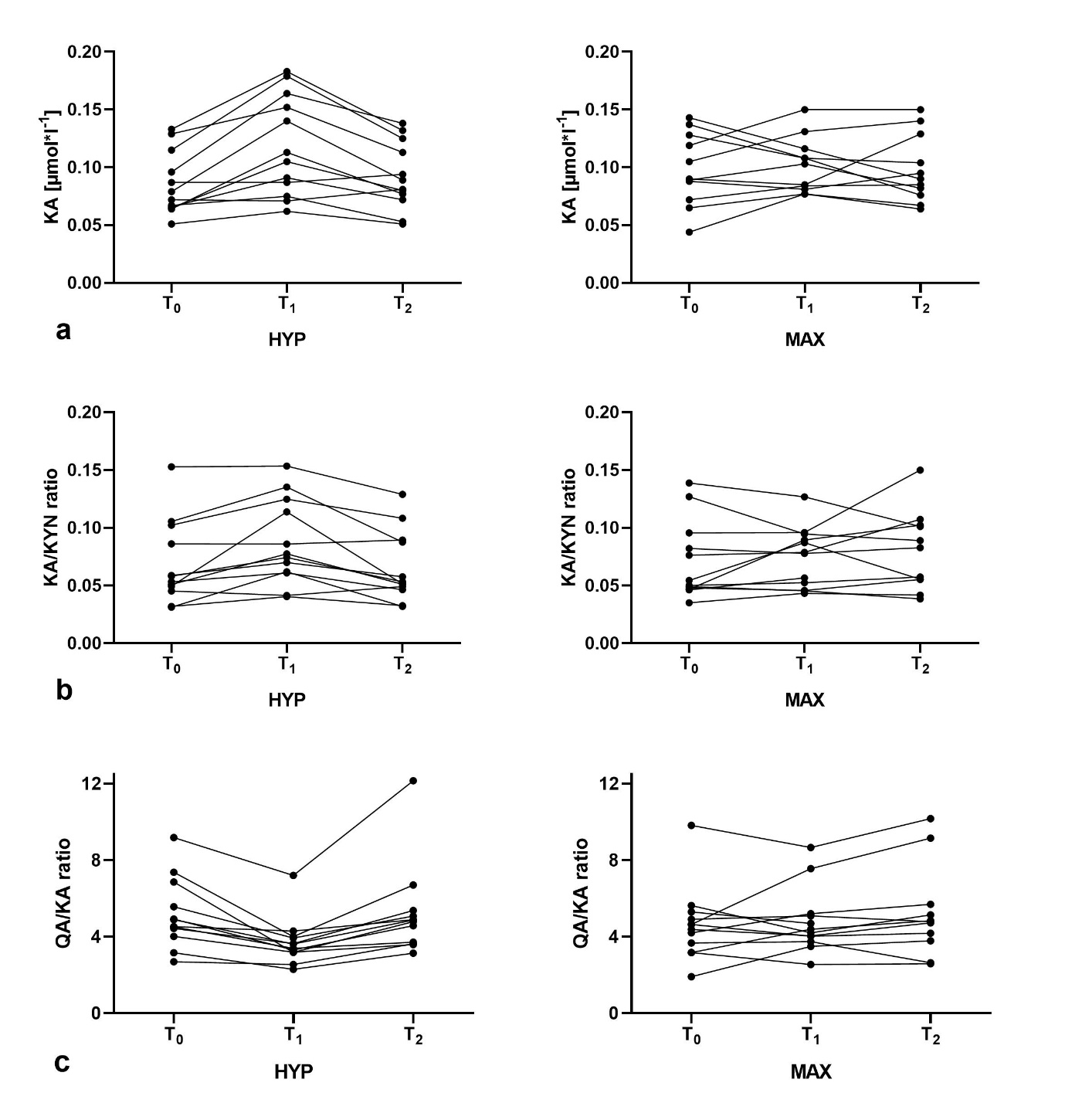


HYP hypertrophic strength loading; MAX maximal strength loading; T0 baseline, T1 immediately after strength loading completion; T2 1h after strength loading completion. (a) kynurenic acid concentrations; (b) kynurenic acid-kynurenine ratio; (c) quinolinic acid-kynurenic acid ratio.
